# Preemptive versus preventive intravenous acetaminophen/ibuprofen fixed-dose combination after robot-assisted radical prostatectomy: a comprehensive secondary analysis of a public double-blind randomized dataset

**DOI:** 10.1007/s11701-026-03239-2

**Published:** 2026-03-06

**Authors:** Hamada Ahmed Youssof, Atef A. Hassan, Ahmed Abdel-Galil Saleh, Ahmed Shafiea, Ahmed Mostafa Mohammed, Ahmed S. Elsayed, Mohamed ElSayed Metwally, Nader A. Abdelkhalek, Gamal M. Hassan, Mohamed Fathy Elebiary, Ahmed Ali Lotfy, Ahmed Sharawy

**Affiliations:** 1 Urology Department, Faculty of Medicine, Fayoum University, Fayoum, Egypt; 2 Faculty of Medicine, Al-Azhar University, Cairo, Egypt; 3 Urology Department, Faculty of Medicine, Al-Azhar University, Cairo, Egypt; 4 Urology Department, Faculty of Medicine, Alexandria University, Alexandria, Egypt; 5 Urology Department, Faculty of Medicine, Cairo University, Cairo, Egypt; 6 Department of Anesthesiology, Faculty of Medicine, Fayoum University, Fayoum, Egypt

**Keywords:** Robot-assisted radical prostatectomy, Multimodal analgesia, Acetaminophen, Ibuprofen, Preventive analgesia, Preemptive analgesia, Opioid-sparing, Quality of recovery, Randomized trial, Secondary analysis

## Abstract

Multimodal, opioid-sparing analgesia is central to enhanced recovery after robot-assisted radical prostatectomy (RALP), yet the incremental value of altering the timing of non-opioid fixed-dose combinations within standardized pathways is uncertain. Using a public, de-identified dataset from a double-blind randomized trial, we performed a comprehensive secondary analysis emphasizing pain burden, trajectories, recovery, and tolerability. Adults undergoing RALP were randomized 1:1 to intravenous acetaminophen 1,000 mg plus ibuprofen 300 mg administered either before incision (preemptive) or at the end of surgery (preventive). The primary endpoint was rest pain burden across 2–48 h, operationalized as the area under the curve (AUC) of numeric rating scale scores. Secondary endpoints included cough pain, opioid consumption, QoR-15 K recovery at 24 h, rescue analgesia, adverse effects, and postoperative day 1 laboratory changes. Linear mixed-effects models analyzed repeated measures. An exploratory elastic-net logistic regression evaluated predictors of high 24-h fentanyl use. Among 152 participants (76 per group), rest pain burden showed no statistically significant between-group difference (mean difference − 6.7, 95% CI − 21.7 to 7.3; Cohen’s d = − 0.14; P = 0.378). Cumulative fentanyl AUC was not significantly different between groups (P = 0.247; SMD = − 0.22). QoR-15 K at 24 h showed no statistically significant difference (mean difference − 3.6, 95% CI − 10.2 to 3.0; P = 0.280). Creatinine change from baseline to postoperative day 1 was similar between groups (P = 0.333). Exploratory prediction of high opioid use (≥ 520 µg) achieved good discrimination (cross-validated AUC 0.84); however, top predictors included postoperative variables, indicating time-ordering leakage that limits prospective clinical utility. Preemptive versus preventive timing of intravenous acetaminophen/ibuprofen was associated with similar observed opioid consumption, rest pain burden, and early recovery within the precision of this sample. These findings are consistent with comparable short-term outcomes across timing strategies; however, the study was not designed as an equivalence trial, and small clinically meaningful differences cannot be excluded.

**Trial registration:** The dataset derives from a double-blind randomized controlled trial registered at ClinicalTrials.gov (NCT05685342). This manuscript reports a secondary analysis of the public de-identified dataset.

**Supplementary Information:**

The online version contains supplementary material available at 10.1007/s11701-026-03239-2.

## Introduction

Robot-assisted radical prostatectomy (RALP) is widely performed for localized prostate cancer within enhanced recovery pathways. Despite minimally invasive techniques, patients commonly experience early postoperative pain from trocar ports, pelvic dissection, urethrovesical anastomosis manipulation, catheter discomfort, and pneumoperitoneum-associated referred pain—factors that can impede mobilization and recovery goals [1, 2].

Opioids effectively treat acute surgical pain but carry risks including nausea, sedation, ileus, and delayed mobilization. Additionally, a subset of opioid-naïve patients transition to persistent use, reinforcing the rationale for opioid-sparing strategies [3, 4]. Enhanced recovery protocols address this by combining scheduled non-opioid analgesics with early mobilization, reserving opioids for rescue [5].

Acetaminophen and NSAIDs form the foundation of multimodal analgesia through complementary mechanisms: acetaminophen provides central analgesia while ibuprofen inhibits cyclooxygenase activity and peripheral sensitization. Fixed-dose intravenous combinations offer additive analgesia with predictable bioavailability when oral intake is limited [6–8].

The timing of analgesic administration, preemptive (before incision) versus preventive (end of surgery), remains debated. Preemptive analgesia aims to attenuate central sensitization, while preventive approaches reduce nociceptive input throughout the perioperative period. Meta-analyses suggest timing effects are modest and context-dependent, necessitating procedure-specific evidence [7].

A double-blind randomized trial comparing preemptive versus preventive intravenous acetaminophen/ibuprofen in RALP has deposited its full de-identified dataset in Harvard Dataverse, enabling transparent secondary analyses [9, 10]. Such shared datasets permit evaluation of outcomes beyond primary reporting, including integrated pain-burden metrics, distributional heterogeneity, and patient-centered recovery measures like the validated QoR-15 instrument [11–13].

We conducted a comprehensive secondary analysis of this public dataset. Our objectives were to quantify postoperative pain burden using an area-under-the-curve framework, characterize pain and opioid trajectories, evaluate early recovery and tolerability, perform sensitivity analyses addressing perioperative imbalances, and conduct exploratory modeling to identify predictors of high opioid requirement.

## Methods

### Study design and data source

This study is a secondary analysis of a public, de-identified dataset deposited in Harvard Dataverse from a double-blind parallel-group randomized trial in adults undergoing RALP under general anesthesia [9]. The trial registration record is available on ClinicalTrials.gov (NCT05685342). The dataset includes allocation group, baseline demographics and comorbidities, perioperative and PACU variables, repeated postoperative pain scores and PCA opioid measures through 48 h, patient-reported QoR-15 K at baseline and 24 h, adverse effects, rescue analgesia indicators, and preoperative and postoperative day 1 laboratory tests.

## Ethics and reproducibility

The dataset is fully de-identified and shared under a CC0 public domain dedication. No attempts were made to re-identify participants or link the dataset to external sources. As the analysis used public non-identifiable data, additional ethics approval was not required. All results reported in this manuscript were generated from reproducible code applied to the deposited spreadsheet with no manual modification of values.

## Participants, Interventions, and analysis population

The analysis population comprised all randomized participants included in the deposited dataset and followed an intention-to-treat principle using the recorded allocation. Participants were randomized 1:1 to receive intravenous acetaminophen 1,000 mg plus ibuprofen 300 mg in 100 mL administered either before incision (preemptive timing) or at the end of surgery (preventive timing), with blinding described in the trial report and registration record [9, 10].

## Outcomes and endpoint definitions

The primary endpoint and analytic plan for this secondary analysis were defined before accessing the public dataset. The prespecified primary endpoint was postoperative rest pain burden across 2–48 h. Pain intensity was recorded on an 11-point NRS (0–10) at 2, 6, 24, and 48 h after surgery. We quantified burden as AUC, computed using the trapezoidal rule by integrating NRS values over time. This approach captures the overall pain experience and reduces dependence on a single time point [14].

Secondary pain endpoints included cough pain at 24 and 48 h and cough pain AUC across 24–48 h. Opioid endpoints included cumulative fentanyl delivered via PCA at 2, 6, 24, and 48 h, summarized at 24 h and as an AUC across 2–48 h; PCA demand was captured as cumulative bolus attempt counts at the same time points and summarized similarly. Patient-reported recovery was assessed using the QoR-15 K at baseline and 24 h; we analyzed both the 24-h score and the change from baseline [11]. Tolerability endpoints were nausea and dizziness recorded at 24 and 48 h. Safety endpoints were changes in AST, ALT, and serum creatinine from baseline to postoperative day 1, consistent with reporting practices for perioperative acetaminophen/NSAID regimens [15, 16].

### Statistical analysis

We summarized continuous variables as mean ± SD when distributions were approximately symmetric and as median [IQR] when skewed; categorical variables were summarized as n (%). Baseline balance was evaluated using standardized mean differences.

For the primary endpoint, we compared rest pain AUC between groups using Welch’s t-test and reported mean differences with 95% confidence intervals. For selected skewed outcomes, we used the Mann–Whitney U test. Binary outcomes were compared using Fisher’s exact test when counts were small. We additionally emphasized effect sizes and distributional overlap using prespecified visualizations.

Repeated measures of rest pain and cumulative fentanyl were analyzed using linear mixed-effects models with random intercepts for participants and fixed effects for group, time (categorical), and group-by-time interaction. For cumulative fentanyl, a log(1 + x) transformation was applied to reduce right-skewness and stabilize variance. Because operative duration differed modestly between groups in the deposited data, we conducted sensitivity analyses adjusting for operative time in (i) a linear regression model for the primary AUC endpoint and (ii) regression for log-transformed 24-h fentanyl; the purpose was to demonstrate robustness rather than to “correct” randomized inference.

For exploratory modeling of high opioid requirement, we defined high 24-h fentanyl use as consumption at or above the 75th percentile of the dataset (≥ 520 µg). Elastic-net logistic regression was fit with standardized predictors and five-fold cross-validation; hyperparameters were chosen by cross-validated AUC. All available baseline, intraoperative, and postoperative variables were included as candidate predictors. Given the exploratory nature and the modest sample size, these models were interpreted as hypothesis-generating [17]. Importantly, because postoperative variables (including pain scores, PCA bolus attempts, adverse effects, and rescue analgesia at various time points) were included in the model, time-ordering leakage was present; consequently, model discrimination reflects associations across the perioperative period rather than true prospective predictive ability from preoperative data alone.

## Results

### Participants and baseline characteristics

A total of 152 participants were randomized equally to preemptive (*n* = 76) or preventive (*n* = 76) groups. Baseline characteristics were well balanced (Table 1). Mean age was 67.9 ± 5.9 versus 67.9 ± 6.2 years (*P* = 0.957), BMI was 24.7 ± 2.5 versus 25.1 ± 2.4 kg/m² (*P* = 0.339), and ASA class was uniformly II. Preoperative QoR-15 K scores were near ceiling in both groups (median 150.0 versus 149.5; *P* = 0.401). Comorbidity prevalence was similar, including hypertension (46.1% versus 53.9%), diabetes (19.7% versus 25.0%), and dyslipidemia (25.0% versus 26.3%; all *P* > 0.4). Baseline laboratory values were comparable.

**Table 1:** Baseline and perioperative characteristics

Variable	Preemptive (*n* = 76)	Preventive (*n* = 76)	*P*	SMD
Demographics				
Age, years	67.9 ± 5.9	67.9 ± 6.2	0.957	−0.01
Weight, kg	68.3 ± 9.2	69.4 ± 7.8	0.412	−0.13
BMI, kg/m²	24.7 ± 2.5	25.1 ± 2.4	0.339	−0.16
ASA class	2.0 [2.0, 2.0]	2.0 [2.0, 2.0]	1.000	0.00
Comorbidities				
Hypertension	35 (46.1%)	41 (53.9%)	0.417	−0.16
Diabetes mellitus	15 (19.7%)	19 (25.0%)	0.559	−0.13
Dyslipidemia	19 (25.0%)	20 (26.3%)	1.000	−0.03
Cardiac disease	1 (1.3%)	2 (2.6%)	1.000	−0.09
Pulmonary disease	5 (6.6%)	1 (1.3%)	0.209	0.27
Hepatic disease	4 (5.3%)	1 (1.3%)	0.367	0.22
Baseline laboratory values				
QoR-15 K	150.0 [144.8, 150.0]	149.5 [143.0, 150.0]	0.401	0.14
AST, U/L	21.0 [19.0, 24.2]	20.5 [18.0, 25.2]	0.946	0.05
ALT, U/L	20.0 [15.8, 29.2]	23.0 [16.8, 28.2]	0.420	0.09
Creatinine, mg/dL	0.9 [0.8, 1.0]	0.9 [0.9, 1.0]	0.950	0.04
Perioperative data				
Operative time, min	95.0 [85.0, 110.0]	90.0 [80.0, 100.0]	0.048	0.29
Anesthesia time, min	121.0 [110.0, 136.2]	117.5 [105.0, 131.0]	0.082	0.26
Remifentanil, µg	500.5 [406.8, 639.5]	495.0 [380.0, 622.8]	0.424	0.06
Remifentanil, µg/kg/hr	3.7 [3.0, 4.4]	3.7 [3.0, 4.4]	0.887	−0.05
PACU fentanyl, µg	0.0 [0.0, 0.0]	0.0 [0.0, 0.2]	0.235	−0.19
Sevoflurane use	65 (85.5%)	63 (82.9%)	0.824	—

Values are mean ± SD, median [IQR], or n (%). SMD = standardized mean difference

## Perioperative characteristics

Operative duration was modestly longer in the preemptive group (median 95.0 versus 90.0 min; *P* = 0.048; SMD = 0.29), though the 5-minute difference was slight (Table 1). Anesthesia time followed a similar pattern (121.0 versus 117.5 min; *P* = 0.082). Intraoperative remifentanil was equivalent (3.7 µg/kg/hr in both groups; *P* = 0.887), PACU fentanyl use was minimal, and volatile anesthetic distribution was similar (sevoflurane 85.5% versus 82.9%; *P* = 0.824).

### Primary endpoint: rest pain burden

**Fig. 1:**
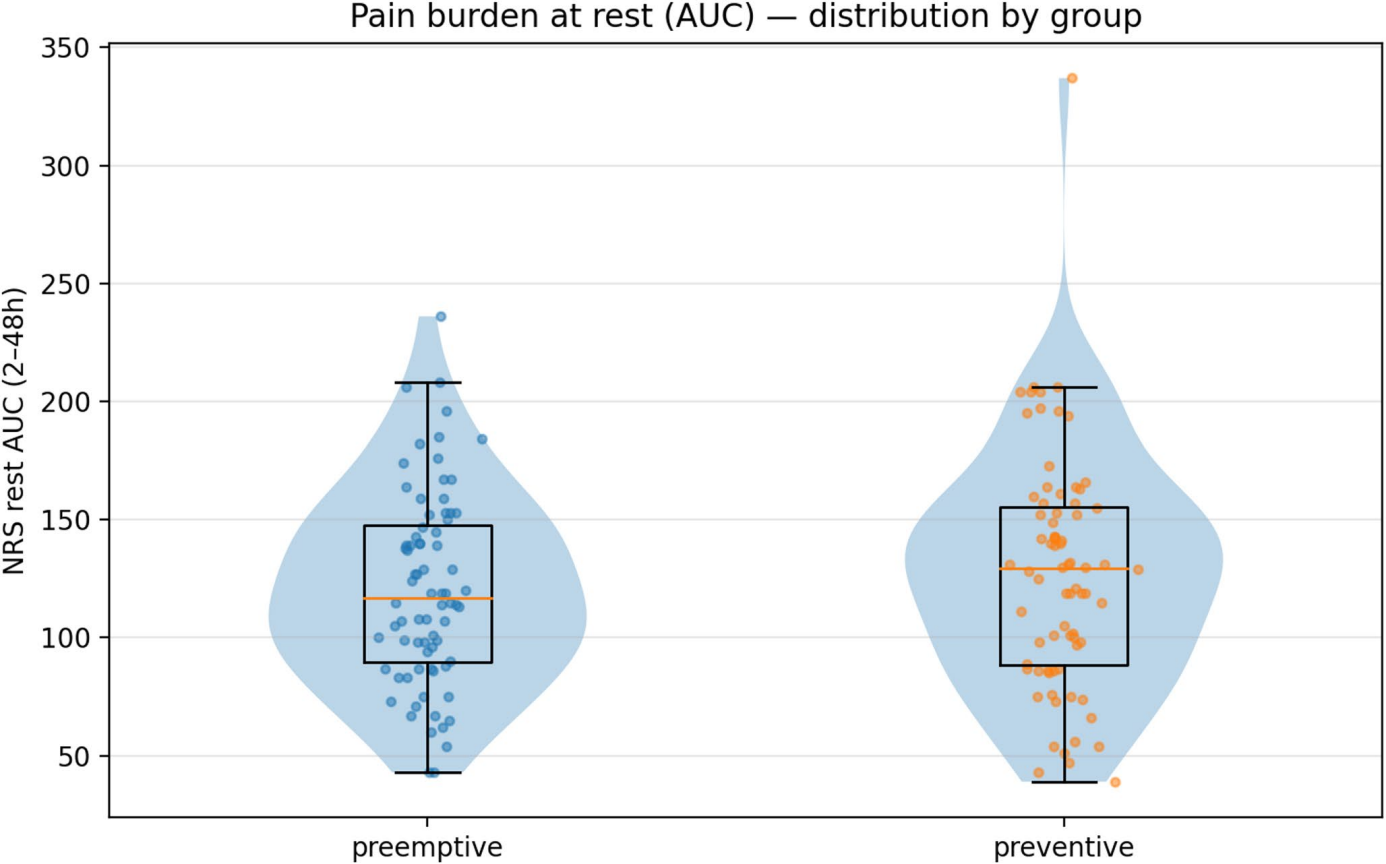
Distribution of rest pain burden (AUC 2–48 h) by group

Rest pain AUC (2–48 h) showed no statistically significant difference between groups (Table 2), though the confidence interval does not exclude clinically relevant effects. Mean AUC was 120.8 ± 41.0 versus 127.4 ± 50.0 NRS·h (*P* = 0.378; Cohen’s d = − 0.14), with a mean difference of − 6.7 (95% CI − 21.7 to 7.3), representing a small standardized effect. Violin plots (Fig. 1) showed extensive overlap in distributions across groups, with nearly identical kernel density profiles, comparable medians (≈ 115–130 NRS·h), and overlapping interquartile ranges. The degree of individual-level overlap suggests that any group-level difference is small relative to within-group heterogeneity.

**Table 2:** Clinical outcomes

Outcome	Preemptive (*n* = 76)	Preventive (*n* = 76)	*P*	SMD
Pain outcomes				
Rest pain AUC (2–48 h), NRS·h	120.8 ± 41.0	127.4 ± 50.0	0.378	−0.14
Cough pain AUC (24–48 h), NRS·h	108.8 ± 31.3	109.3 ± 36.0	0.931	−0.01
Opioid consumption				
Fentanyl AUC (2–48 h), µg·h	11,400 [6,275–21,120]	15,020 [7,190–25,050]	0.247	−0.22
PCA attempts AUC (2–48 h)	852 [488–1,612]	1,086 [512–2,304]	0.348	−0.10
Quality of recovery				
QoR-15 K at 24 h	111.0 ± 20.7	114.6 ± 20.4	0.280	−0.18
QoR-15 K change from baseline	−35.5 ± 19.8	−31.1 ± 19.7	0.169	−0.22
Rescue analgesia				
At 2 h	1 (1.3%)	2 (2.6%)	1.000	−0.09
At 6 h	0 (0%)	3 (3.9%)	0.245	−0.28
At 24 h	3 (3.9%)	4 (5.3%)	1.000	−0.06
At 48 h	3 (3.9%)	2 (2.6%)	1.000	0.07
Adverse effects				
Nausea at 24 h	11 (14.5%)	5 (6.6%)	0.186	0.26
Dizziness at 24 h	7 (9.2%)	8 (10.5%)	1.000	−0.04
Nausea at 48 h	3 (3.9%)	3 (3.9%)	1.000	0.00
Dizziness at 48 h	3 (3.9%)	3 (3.9%)	1.000	0.00
Laboratory safety (change from baseline)				
AST, U/L	−0.6 ± 6.3	−0.7 ± 5.3	0.934	0.01
ALT, U/L	−4.0 [−7.0, −1.0]	−4.0 [−6.2, −1.0]	0.928	−0.11
Creatinine, mg/dL	−0.1 [−0.1, −0.0]	−0.1 [−0.1, −0.0]	0.333	−0.16

Pain burden expressed as area under the NRS-time curve (NRS·h). Opioid exposure as cumulative area under the curve (µg·h). Laboratory values as postoperative day 1 minus baseline

### Pain trajectories

**Fig. 2:**
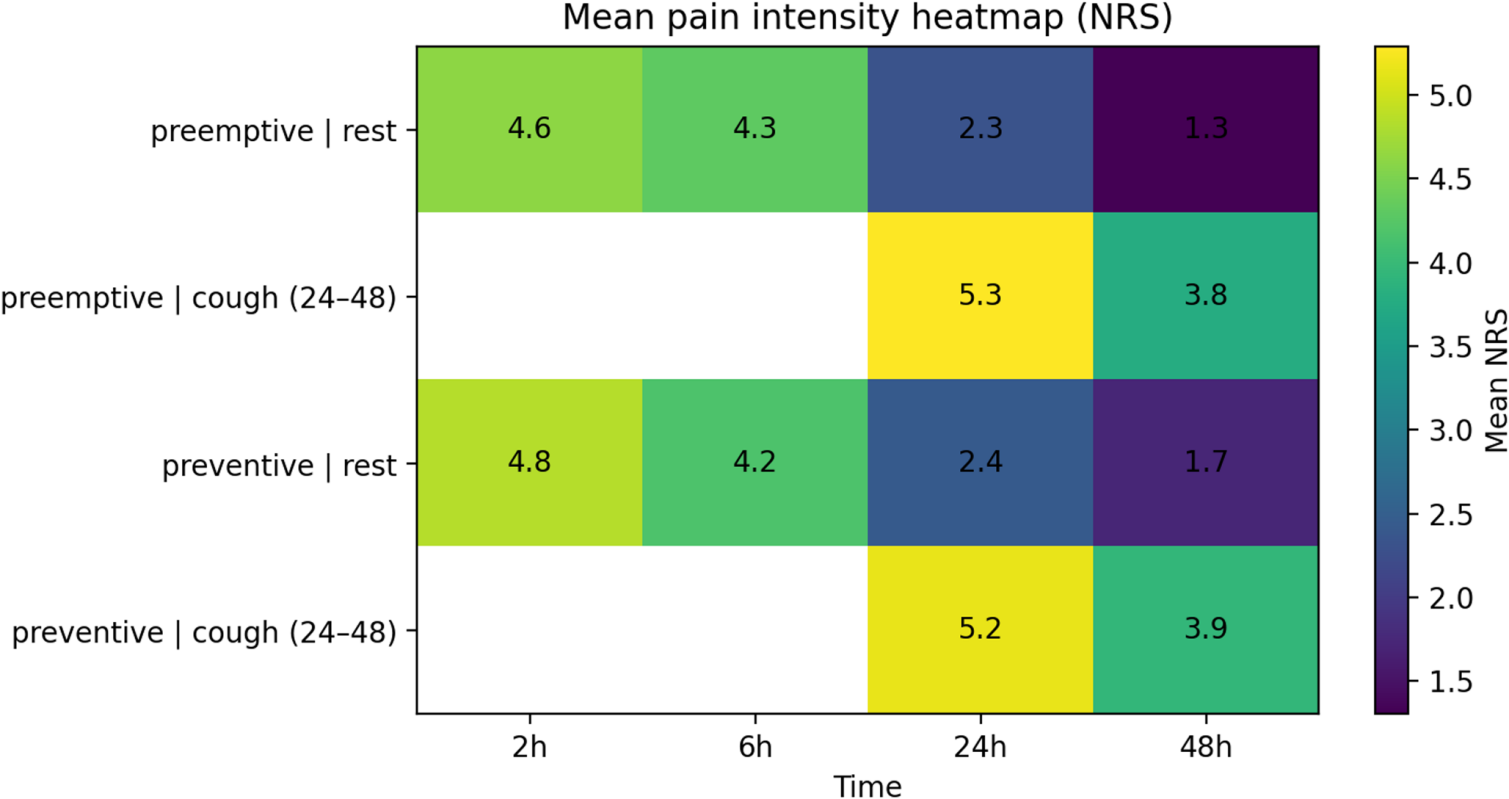
Pain intensity trajectories (Heatmap)

The heatmap (Fig. 2) displays pain trajectories. Rest pain declined from 4.6 versus 4.8 at 2 h to 1.3 versus 1.7 at 48 h, with the most pronounced decline between 6 and 24 h. Cough pain was higher than rest pain: 5.3 versus 5.2 at 24 h, declining to 3.8 versus 3.9 at 48 h. Cough pain AUC was nearly identical (108.8 ± 31.3 versus 109.3 ± 36.0; *P* = 0.931).

Mixed-effects modeling (Table 3) confirmed significant time effects (24 h: coefficient − 2.29, *P* < 0.001; 48 h: coefficient − 3.30, *P* < 0.001) but no group effect (coefficient 0.24; *P* = 0.226) or significant group-by-time interactions.

**Table 3:** Mixed-effects model for rest pain over time

Term	Coefficient	SE	z	*P*
Intercept	4.61	0.14	33.31	< 0.001
Preventive group	0.24	0.20	1.21	0.226
Time 6 h	−0.30	0.18	−1.69	0.092
Time 24 h	−2.29	0.18	−12.76	< 0.001
Time 48 h	−3.30	0.18	−18.41	< 0.001
Preventive × 6 h	−0.37	0.25	−1.45	0.147
Preventive × 24 h	−0.12	0.25	−0.47	0.641
Preventive × 48 h	0.18	0.25	0.73	0.468
Random intercept variance	0.23	0.06	—	—

Linear mixed-effects model with random intercept for participant. Reference category: preemptive group at 2 h

### Opioid consumption

**Fig. 3:**
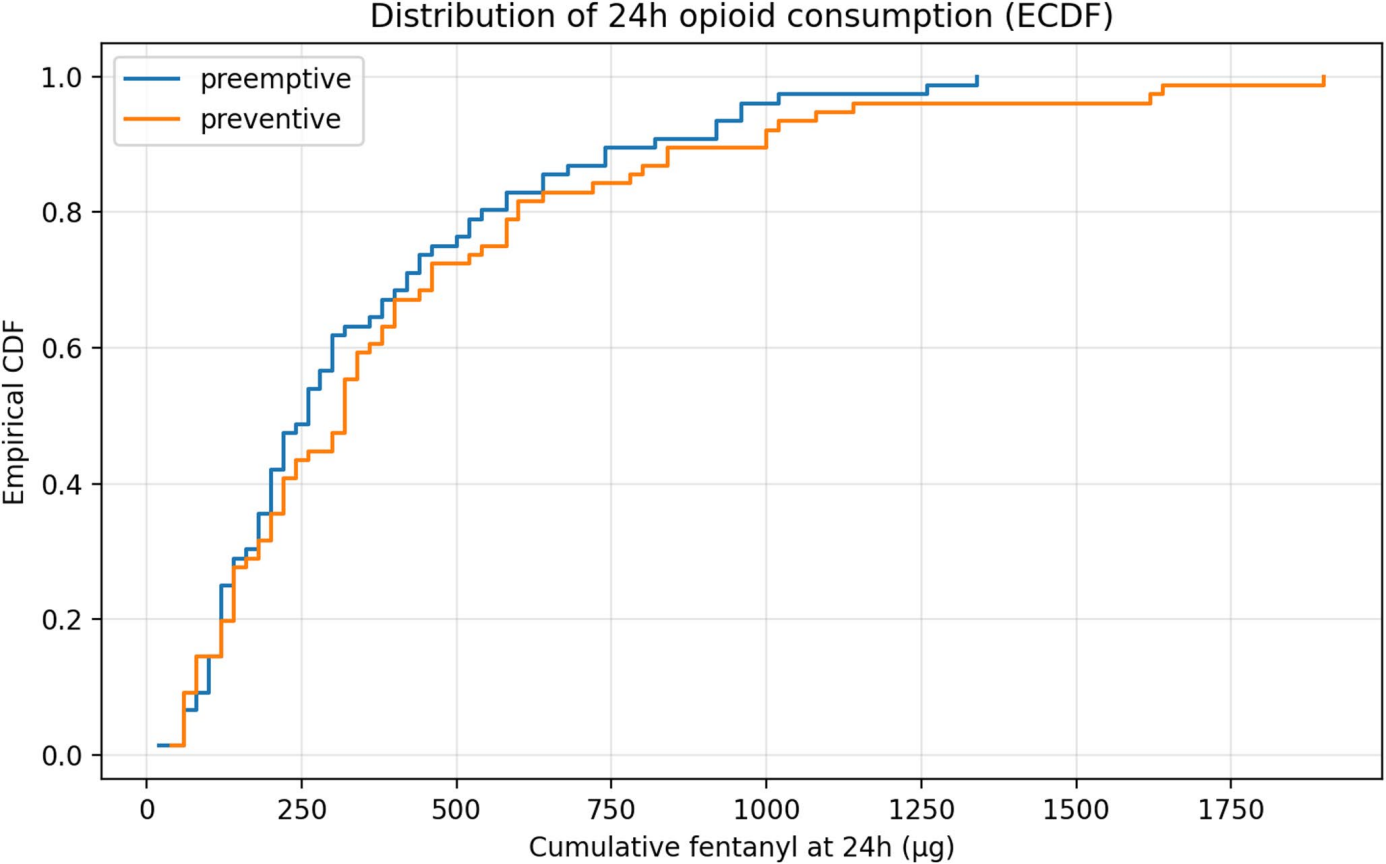
Cumulative distribution of 24-hour fentanyl consumption

Cumulative fentanyl AUC showed no statistically significant between-group difference (Table 2): median 11,400 versus 15,020 µg·h (*P* = 0.247; SMD = − 0.22), although the wide confidence interval precludes definitive conclusions. PCA bolus attempts were also similar (852 versus 1,086; *P* = 0.348). The empirical cumulative distribution functions (Fig. 3) were largely superimposable across the full range of consumption (0–1,900 µg).

Mixed-effects modeling (**Supplementary Table S1**) showed no main group effect (*P* = 0.920). A significant group-by-time interaction at 48 h (coefficient 0.27; *P* = 0.003) indicated divergent patterns of late accumulation. Back-transformed estimated means at 24 h were 255 versus 284 µg (**Supplementary Table S2**).

### Quality of recovery

QoR-15 K at 24 h showed no statistically significant between-group difference (Table 2): 111.0 ± 20.7 versus 114.6 ± 20.4 (*P* = 0.280; Cohen’s d = − 0.18; mean difference − 3.6 [95% CI − 10.2 to 3.0]). Change from baseline was − 35.5 ± 19.8 versus − 31.1 ± 19.7 points (*P* = 0.169). Both differences were below the minimal clinically significant difference of 8 points. The paired-trajectory plot (**Supplementary Figure S1**) shows similar decline patterns, with considerable individual heterogeneity.

### Rescue analgesia and adverse effects

Rescue analgesia was infrequent and similar between groups (Table 2): cumulative use reached 8% versus 11% by 48 h. Cumulative incidence curves (**Supplementary Figure S2**) showed numerically higher early rescue use in the preventive group, though confidence intervals overlapped.

Nausea at 24 h was numerically more frequent in the preemptive group (14.5% versus 6.6%; *P* = 0.186), while dizziness was similar (9.2% versus 10.5%; *P* = 1.000). By 48 h, both adverse effects were identical (3.9% each).

### Laboratory safety

Postoperative day 1 laboratory changes were comparable and clinically unremarkable (Table 2). AST change was − 0.6 ± 6.3 versus − 0.7 ± 5.3 U/L (*P* = 0.934). Creatinine change was median − 0.1 mg/dL in both groups (*P* = 0.333). The scatter plot (**Supplementary Figure S3**) shows no baseline-dependent differential effects.

### Effect-Size summary

**Fig. 4:**
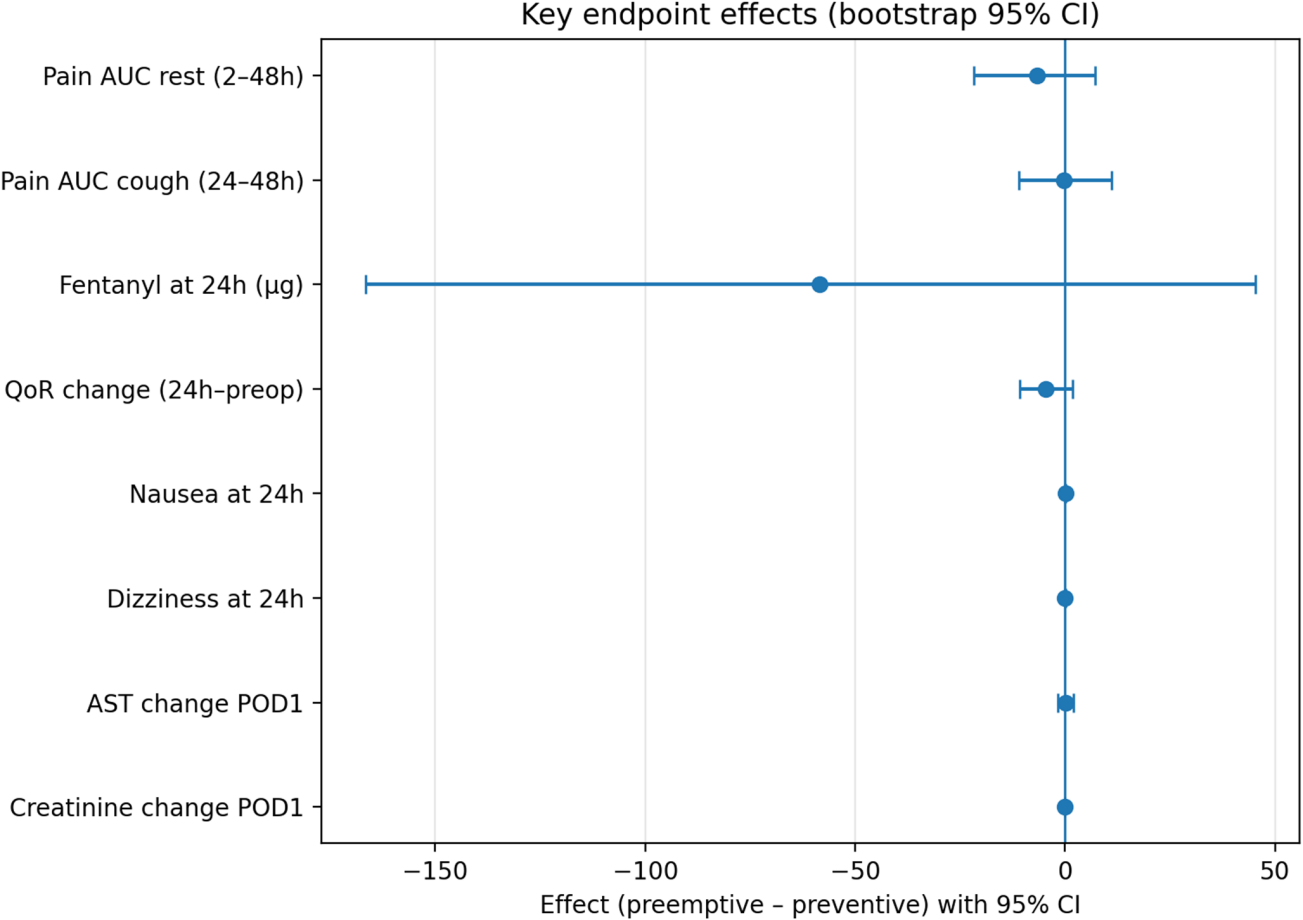
Effect-size forest plot

The forest plot (Fig. 4) synthesizes effect estimates. All confidence intervals crossed zero: rest pain AUC − 6.7 (95% CI −21.7 to 7.3), cough pain AUC − 0.3 (−10.9 to 11.1), fentanyl at 24 h −58.4 µg (−166.6 to 45.3), QoR change − 4.7 (−10.8 to 1.8), nausea risk difference 0.08 (−0.01 to 0.18), and creatinine change − 0.02 (−0.06 to 0.02).

### Exploratory prediction of high opioid use

High 24-hour fentanyl use (≥ 520 µg) occurred in 39 participants (25.7%). Elastic-net logistic regression achieved an AUC of 0.84 (Table 4). Top predictors included bolus attempts at 48 h (coefficient 2.37), bolus attempts at 24 h (2.06), cough pain at 48 h (0.44), diabetes (−0.40), and age (−0.31).

**Table 4:** Exploratory predictive model for high 24-Hour Fentanyl use

Model performance		
Cross-validated AUC	0.84	
Sample size	152	
High-use events	39 (25.7%)	
Threshold	520 µg	

Top predictors	Coefficient	Variable timing
Bolus attempts 48 h	2.37	Post-outcome
Bolus attempts 24 h	2.06	Contemporaneous
Cough pain 48 h	0.44	Post-outcome
Diabetes mellitus	−0.40	Baseline
Sevoflurane use	0.36	Intraoperative
Pulmonary disease	−0.36	Baseline
Rest pain 24 h	0.33	Contemporaneous
Dizziness 24 h	0.31	Contemporaneous
Age	−0.31	Baseline
Dyslipidemia	−0.30	Baseline
QoR-15 K preoperative	0.23	Baseline

Note: Coefficients represent penalized log-odds per standard deviation increase in each predictor; positive values indicate higher probability of high opioid use, and negative values indicate lower probability. Because elastic-net applies regularization, coefficients are shrunk toward zero, and their magnitudes reflect relative rather than absolute importance. Model includes time-ordering leakage from variables measured at or after the outcome timepoint, limiting clinical utility for prospective prediction. This model should not be interpreted as a clinically deployable predictive tool. Elastic-net logistic regression with 5-fold cross-validation. High use defined as ≥520 µg (75th percentile)

However, the model included postoperative variables (bolus attempts, pain scores, dizziness at 24–48 h), representing time-ordering leakage. The high discrimination reflects temporal correlations rather than genuine prospective predictive ability. Among baseline predictors alone, diabetes, pulmonary disease, age, and preoperative QoR-15 K showed modest associations, suggesting limited predictive utility from preoperative data.

## Discussion

In this secondary analysis of a public double-blind randomized dataset, preemptive versus preventive timing of intravenous acetaminophen/ibuprofen in RALP was associated with similar observed rest pain burden (mean difference − 6.7; 95% CI − 21.7 to 7.3), opioid consumption, and patient-reported recovery. Point estimates for all efficacy, tolerability, and safety endpoints were small; however, confidence intervals were sufficiently wide that clinically meaningful differences in either direction cannot be excluded.

Our findings of similar outcomes across timing strategies are consistent with a body of evidence questioning the magnitude of preemptive analgesia effects, although timing effects may be context-dependent, procedure-specific, and agent-specific rather than universally absent. Møiniche et al. systematically reviewed 80 trials and concluded that preemptive analgesia failed to demonstrate consistent benefits, catalyzing a conceptual shift in perioperative pain management [18]. Ong et al. subsequently analyzed 66 RCTs and found that while preemptive NSAIDs modestly improved analgesic consumption, they did not improve pain scores compared with postincisional administration [7]. Dahl and Kehlet recommended abandoning “pre-emptive” terminology, emphasizing that duration and efficacy of multimodal coverage matter more than precise incision timing [19].

For minimally invasive surgery, Coughlin et al. meta-analyzed 26 laparoscopic surgery RCTs and found no timing effect for incision-site infiltration [20]. In RALP specifically, Sisa et al. demonstrated no benefit of preemptive pregabalin when multimodal analgesia was employed (24-hour consumption: 15 versus 17 mg morphine equivalents; *P* = 0.44), consistent with our findings using a different non-opioid agent [21]. Notably, the same intravenous acetaminophen/ibuprofen fixed-dose combination demonstrated significant opioid-sparing effects in video-assisted thoracic surgery, reducing consumption by 100–140 µg fentanyl equivalent at 24–48 h compared with placebo [22], confirming the analgesic efficacy of this combination even though timing effects remain negligible.

The 24-hour fentanyl consumption observed (255–284 µg, approximately 25–28 mg morphine equivalents) falls within established RALP benchmarks. Ashrafi et al. demonstrated 67% opioid reduction with aggressive ERAS implementation (15 versus 46 mg morphine equivalents) [23], while Taninishi et al. reported 200–210 µg fentanyl with TAP blocks. Our results represent adequate systemic multimodal analgesia without regional techniques [24]. Lee et al. showed that a comprehensive non-opioid multimodal protocol combining pregabalin, paracetamol, and regional blocks was non-inferior to morphine-based PCA for RALP, with 58% of patients requiring no opioids for 48 h [25]. Our results represent adequate systemic multimodal analgesia without regional techniques.

The PROSPECT guidelines recommend scheduled paracetamol and NSAIDs as foundational analgesia for prostatectomy [1]. Importantly, ERAS protocols typically involve scheduled repeated dosing of non-opioid analgesics, often initiated orally hours before surgery and continued postoperatively. Our analysis examines only the timing of a single intravenous fixed-dose combination within a standardized anesthetic protocol; consequently, these findings should not be extrapolated to the broader question of scheduled multimodal analgesic timing within comprehensive ERAS pathways. Within the scope of single-dose timing, the effect may be subtle and difficult to detect without larger samples. The fixed-dose combination itself has robust efficacy evidence: Daniels et al. demonstrated superior analgesia compared with monotherapy [6], and a meta-analysis by Abushanab and Al-Badriyeh confirmed a relative risk of 2.60 for achieving ≥ 50% pain relief versus placebo [26].

The QoR-15 K decline (31–35 points) mirrors that observed in the Korean validation study (4), which reported a median decrease of 36.5 points. This represents approximately four times the established MCID of 8 points [13], indicating expected deterioration consistent with major surgery. The between-group point estimate (− 4.4 points; 95% CI − 10.8 to 1.8) fell below this threshold; however, the upper bound of the confidence interval (− 10.8) exceeds the MCID of 8 points in absolute value, indicating that a clinically meaningful difference in favor of preventive timing cannot be excluded with certainty.

The absence of renal or hepatic signal is consistent with the Lee et al. Cochrane review, which found NSAIDs cause only clinically unimportant transient changes in renal function among adults with normal baseline values [15]. These findings are consistent with a comparable short-term safety profile across timing strategies within this sample, though the study was not powered to detect rare adverse events.

The clinical significance of opioid-sparing extends beyond acute hospitalization. Brummett et al. established that 5.9–6.5% of opioid-naïve surgical patients develop persistent use [3] Santosa et al. demonstrated an associated hazard ratio for mortality of 3.44. For prostatectomy specifically, rates are lower (< 1–1.3%), but minimizing exposure remains prudent [27].

This analysis operationalized pain burden using an AUC framework integrating multiple assessments [14], with distributional visualizations clarifying heterogeneity. To contextualize the clinical meaning of the primary AUC metric, the observed 6.7 NRS·h difference over the 46-hour observation window corresponds to approximately 0.15 NRS points averaged across all time points—a magnitude well below the threshold patients can typically perceive. The exploratory prediction model achieved high discrimination (AUC 0.84) but included postoperative variables representing time-ordering leakage. Critically, this model should not be interpreted as a clinically deployable predictive tool; it serves only as an exploratory association analysis identifying variables correlated with high opioid consumption across the perioperative period. Among baseline predictors, associations were modest, suggesting that clinically useful prospective prediction from preoperative data remains elusive and would require dedicated prediction studies with appropriate selection of temporal variables.

### Limitations

Several limitations warrant consideration. First, and most importantly, this study was designed as a superiority trial and was neither designed nor powered as an equivalence or non-inferiority trial; no equivalence margin was prespecified. Consequently, the absence of a statistically significant difference does not establish that the two timing strategies produce equivalent outcomes, and clinically meaningful differences in either direction cannot be excluded. Second, follow-up was limited to 48 h, precluding evaluation of persistent opioid use and longer-term recovery. Third, the sample size (*n* = 152) may be insufficient for detecting small but meaningful differences. Fourth, this single-centre Korean dataset may limit generalizability. Fifth, the modest operative duration imbalance (95 versus 90 min; *P* = 0.048), though addressed in sensitivity analyses, represents imperfect randomization. Sixth, the analysis evaluates only the timing of a single intravenous fixed-dose combination, whereas ERAS protocols typically employ scheduled repeated multimodal analgesic dosing beginning preoperatively; therefore, these single-dose findings cannot be generalized to the timing effects of scheduled multimodal regimens within comprehensive ERAS pathways. Seventh, regional techniques were not employed; PROSPECT recommends TAP blocks as the first-choice regional technique, which might modify timing effects. Eighth, near-ceiling preoperative QoR-15 K scores may limit sensitivity to detect differences. Finally, the exploratory prediction model included time-ordering leakage, which limits its clinical applicability.

### Future directions

Several avenues merit further investigation. First, larger multicenter trials with extended follow-up (≥ 90 days) are needed to evaluate whether analgesic timing influences persistent opioid use, chronic postsurgical pain, and long-term functional recovery.

Second, factorial designs comparing timing effects across different non-opioid combinations (e.g., acetaminophen/ibuprofen versus acetaminophen/ketorolac versus acetaminophen/COX-2 inhibitors) would clarify whether the null timing effect is agent-specific or generalizable.

Third, studies incorporating regional techniques should examine whether systemic analgesic timing interacts with regional blockade effectiveness.

Fourth, patient stratification based on preoperative pain sensitivity phenotyping, psychological factors, or genetic polymorphisms affecting drug metabolism may identify subgroups who differentially benefit from optimized timing.

Fifth, implementation research examining how timing flexibility affects perioperative workflow efficiency, medication safety, and protocol adherence in high-volume centers would translate these findings into practice.

Sixth, primary research comparing the timing effects of scheduled multimodal analgesic regimens, including oral preoperative loading and postoperative continuation, within comprehensive ERAS protocols for RALP is needed to determine whether timing effects differ when non-opioid analgesics are administered as part of a scheduled dosing strategy rather than as a single perioperative dose.

Finally, incorporating patient-reported experience measures beyond the QoR-15, including satisfaction with pain control, sleep quality, and return to baseline activities, would provide a more comprehensive assessment of recovery quality.

## Conclusions

Preemptive versus preventive administration of intravenous acetaminophen/ibuprofen was associated with similar observed outcomes across all endpoints within the precision of this sample. These findings are consistent with evidence questioning the magnitude of preemptive analgesia timing effects; however, as this study was not designed as an equivalence trial, clinically meaningful differences cannot be excluded. Adequately powered equivalence or non-inferiority trials using scheduled multimodal dosing regimens are needed before timing flexibility can be formally endorsed.

## Supplementary Information

Below is the link to the electronic supplementary material.

Supplementary Material 1

## Data Availability

The dataset analyzed in this study is publicly available through Harvard Dataverse (DOI: 10.7910/DVN/GDW6XG).

## Code availability

The analysis code used to generate the results is available from the corresponding author upon reasonable request.
