# Supplementary material for: Preemptive versus preventive intravenous acetaminophen/ibuprofen fixed-dose combination after robot-assisted radical prostatectomy: a comprehensive secondary analysis of a public double-blind randomized dataset
Source: J Robot Surg. 2026 Mar 6;20(1):320. doi: 10.1007/s11701-026-03239-2 (PMC12966217; doi:10.1007/s11701-026-03239-2)
Supplement: Supplementary file 1 — Supplementary Material 1 [file 11701_2026_3239_MOESM1_ESM.docx]

# **Supplementary Materials**


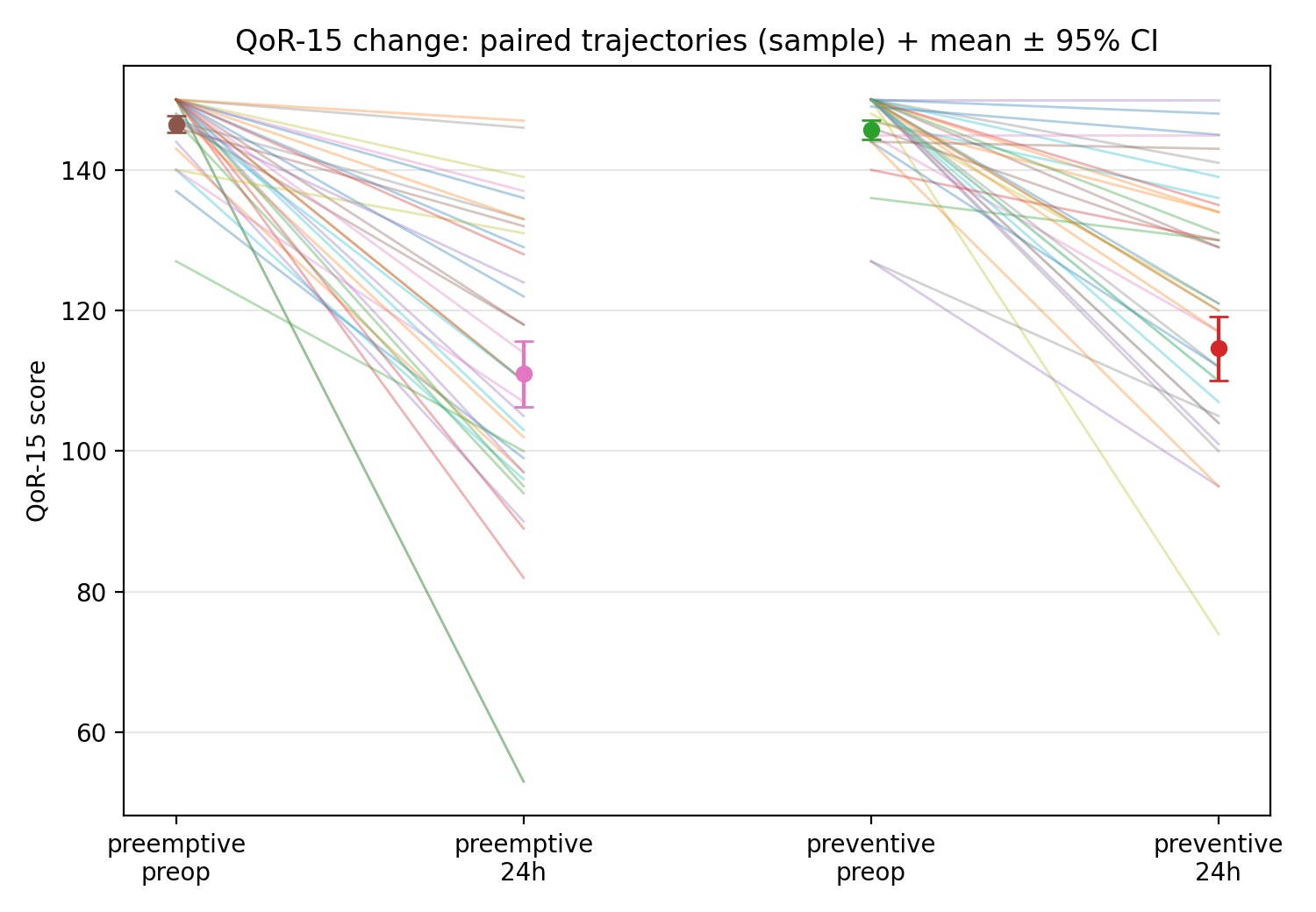


## **Supplementary Figure S**1**. Quality of Recovery Trajectories**

Paired slope plot showing individual QoR-15K trajectories from preoperative to 24 hours for a random sample, with group means and 95% confidence intervals. Baseline scores clustered near the ceiling (~146–150) in both groups. At 24 hours, scores declined to approximately 111 (preemptive, pink marker) and 115 (preventive, red marker). Individual trajectories show heterogeneous decline patterns, with similar distributions between groups.


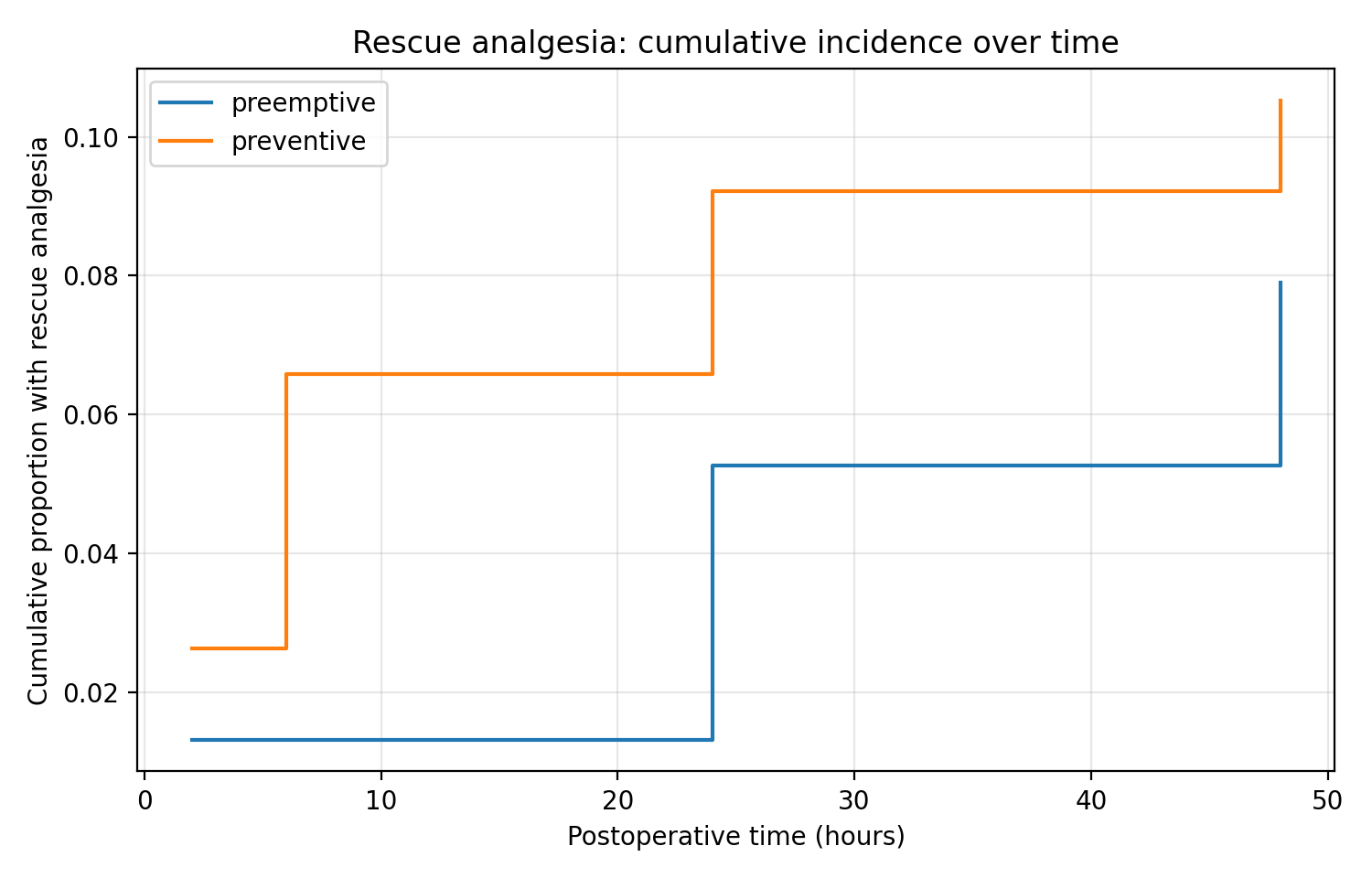


## **Supplementary Figure S**2**. Cumulative Incidence of Rescue Analgesia**

Step functions showing the cumulative proportion of participants receiving rescue analgesia by postoperative time. The preventive group (orange) shows earlier and higher rescue use, reaching approximately 2.6% at two h, 6.6% at six h, 9.2% at 24 h, and 10.5% at 48 h. The preemptive group (blue) shows lower cumulative rescue use: 1.3% at two h, 1.3% at six h, 5.3% at 24 h, and 7.9% at 48 h.


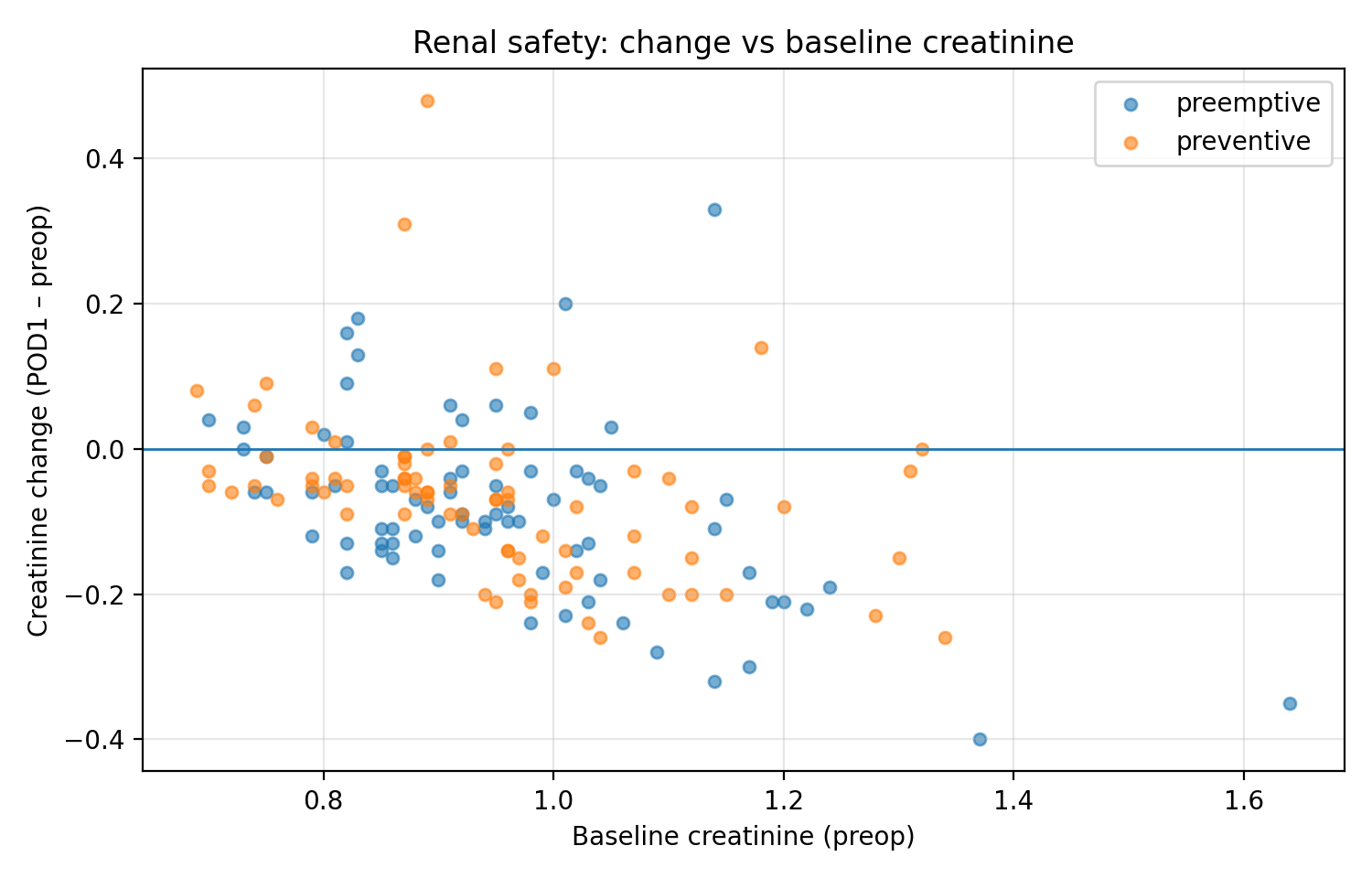


## **Supplementary Figure S**3**. Renal Safety: Creatinine Change Versus Baseline**

Scatter plot of creatinine change (postoperative day 1 minus preoperative) versus baseline creatinine by group (preemptive: blue; preventive: orange). Points are intermixed with no systematic separation. Most participants showed small decreases in creatinine (below the zero reference line). No baseline-dependent differential effects were observed across the range of baseline creatinine (0.65–1.65 mg/dL).

## **Supplementary Table S1. Mixed-Effects Model for Cumulative Fentanyl Over Time (Log Scale)**

| Term | Coefficient | SE | z | P | 95% CI |
| --- | --- | --- | --- | --- | --- |
| Intercept | 4.34 | 0.09 | 49.46 | <0.001 | 4.16, 4.51 |
| Preventive group | -0.01 | 0.12 | -0.10 | 0.920 | -0.26, 0.23 |
| Time 6 h | 0.66 | 0.06 | 10.44 | <0.001 | 0.54, 0.78 |
| Time 24 h | 1.21 | 0.06 | 19.20 | <0.001 | 1.09, 1.34 |
| Time 48 h | 1.51 | 0.06 | 23.99 | <0.001 | 1.39, 1.64 |
| Preventive × 6 h | 0.01 | 0.09 | 0.13 | 0.897 | -0.16, 0.19 |
| Preventive × 24 h | 0.12 | 0.09 | 1.33 | 0.185 | -0.06, 0.29 |
| Preventive × 48 h | 0.27 | 0.09 | 3.02 | 0.003 | 0.10, 0.44 |
| Random intercept variance | 0.43 | 0.16 | — | — | — |

Linear mixed-effects model for log(1+fentanyl) with random intercept for participant.

## **Supplementary Table S2. Model-Estimated Marginal Means**

Rest Pain (NRS)

| Time | Preemptive | Preventive |
| --- | --- | --- |
| 2 h | 4.61 | 4.84 |
| 6 h | 4.30 | 4.17 |
| 24 h | 2.32 | 2.43 |
| 48 h | 1.30 | 1.72 |

Cumulative Fentanyl (µg, back-transformed from log scale)

| Time | Preemptive | Preventive |
| --- | --- | --- |
| 2 h | 75 | 74 |
| 6 h | 146 | 146 |
| 24 h | 255 | 284 |
| 48 h | 345 | 447 |
